# Functional Brain Changes Following Burn Injury: A Narrative Review

**DOI:** 10.1177/15459683231215331

**Published:** 2023-12-03

**Authors:** Grant Rowe, Amira Allahham, Dale W. Edgar, Brittany K. Rurak, Mark W. Fear, Fiona M. Wood, Ann-Maree Vallence

**Affiliations:** 1 School of Psychology, College of Health and Education, Murdoch University, Murdoch, WA, Australia; 2 Burn Injury Research Unit, School of Biomedical Sciences, University of Western Australia, Crawley, WA, Australia; 3 Fiona Wood Foundation, Murdoch, WA, Australia; 4 Burn Service of Western Australia, Fiona Stanley Hospital, MNH (B) Main Hospital, Level 4, Burns Unit, Murdoch, WA, Australia; 5 Institute for Health Research, The University of Notre Dame Australia, Fremantle, WA, Australia; 6 Centre for Healthy Ageing, Health Futures Institute, Murdoch University, Murdoch, WA, Australia; 7 Centre for Molecular Medicine and Innovative Therapeutics, Health Futures Institute, Murdoch University, Murdoch, WA, Australia

**Keywords:** burn injury, central nervous system, brain, non-invasive brain stimulation, neuroplasticity, burn rehabilitation

## Abstract

**Background:**

Burn injuries cause significant motor and sensory dysfunctions that can negatively impact burn survivors’ quality of life. The underlying mechanisms of these burn-induced dysfunctions have primarily been associated with damage to the peripheral neural architecture, however, evidence points to a systemic influence of burn injury. Central nervous system (CNS) reorganizations due to inflammation, afferent dysfunction, and pain could contribute to persistent motor and sensory dysfunction in burn survivors. Recent evidence shows that the capacity for neuroplasticity is associated with self-reported functional recovery in burn survivors.

**Objective:**

This review first outlines motor and sensory dysfunctions following burn injury and critically examines recent literature investigating the mechanisms mediating CNS reorganization following burn injury. The review then provides recommendations for future research and interventions targeting the CNS such as non-invasive brain stimulation to improve functional recovery.

**Conclusions:**

Directing focus to the CNS following burn injury, alongside the development of non-invasive methods to induce functionally beneficial neuroplasticity in the CNS, could advance treatments and transform clinical practice to improve quality of life in burn survivors.

## Introduction

The prevalence of burn injuries is a global community health problem: an estimated 11 million medically reported burn incidences and more than 180 000 burn-related deaths are reported annually.^1^ Treatment approaches for burn injuries are strongly influenced by burn size and depth^2^ and although treatment innovation has seen patient life expectancy improve over the years, burn injuries still cause significant physiological and psychological harm that can negatively impact quality of life.^3^ Motor and sensory dysfunction, including pain, itch, and hypersensitivity, are the main functional impairments that impact burn survivors’ quality of life.^3,4^ Although there are abundant reports about these burn-induced dysfunctions, the mechanisms driving these effects are not well understood. Given the systemic inflammation,^5^ afferent dysfunction,^6^ and persistent pain^7^ that occurs following burn injury, and that cortical^8^ and spinal cord^9^ plasticity have been subsequently detected, it is plausible there are centrally mediated functional impairments because of burn injury. If functional changes in the brain contribute to burn-induced functional impairments, then treatment approaches that specifically target the central nervous system (CNS) could transform clinical practice for burn care. The aim of this narrative review on functional brain changes following burn injury is to critically examine functional impairments in the motor and sensory systems and the mechanisms that could mediate cortical reorganization following burn injury. The review also offers recommendations for future research using interventions targeting the CNS in burn treatment and rehabilitation.

## Functional Impairments Following Burn Injury

### Motor Dysfunction

Both severe and non-severe burn injuries are associated with prolonged motor dysfunction that can lead to increased risk of permanent movement maladaptations becoming resistant to treatments.^2^ Following severe burn injury (total body surface area [TBSA] = 51%), joint pain and stiffness, problems with gait, fatigue, and weak arms and hands are reported up to 17 years post-injury.^10^ Following non-severe burn injury (TBSA = 8.1%), self-reported upper-limb motor dysfunction is associated with higher rates of disability 12 months post-injury.^11^ Similar outcomes are observed in children with both severe (TBSA > 20%) and non-severe (TBSA < 20%) burn injuries affecting the hands, with parents reporting both fine and gross motor function deficits in the 5 years after injury.^12^ Burn depth also contributes to motor dysfunction, with full thickness injuries associated with greater upper-limb disability than other burn types such as partial thickness and superficial burns.^11^ Deeper burns are associated with increased scar^13^ which results in decreased range of movement^14^ and sensation,^15^ likely leading to greater functional impairment. Deeper burns are also treated with surgery,^16^ and thus, surgical trauma can cause further motor function challenges. Collectively, these findings highlight that severe and non-severe burn injuries can cause significant motor impairment in children and adults. The resultant motor dysfunction negatively affects a burn survivors’ ability to carry out tasks of daily living, which in turn, negatively affects their capacity to return to work and perform caring responsibilities.^17^

### Cutaneous Sensory Dysfunction

Sensory function in the scar tissue is significantly reduced compared to skin tissue at a homologous non-injured site,^15^ even after minor burns that have good quality scars, defined as flatter scars with vascularity, pigmentation, and pliability normalized to the individual.^18^ Sensory function in non-injured skin tissue in burn survivors is also reduced compared to non-injured controls.^19^ This disrupted sensory function may be associated with chronic pain and chronic itch for decades post-injury, with pain in ~52% of burn survivors^20^ and itch in ~44% of burn survivors.^21^ Self-report measures of health and quality of life show that burn survivors who suffered recent burns (<12 months) experience heat sensitivity (Burn Specific Health Scale-Brief), bodily pain (Short Form-36 [SF-36]), and pain/discomfort (EuroQol questionnaire), while long-term burn survivors also experience emotional functioning challenges (SF-36).^3^ Self-report measures of itch (Patient and Observer Scar Assessment) are also negatively associated with mental health (SF-36) over a 6-month period following injury.^22^ These findings highlight the negative association of sensory dysfunction with burn survivors’ restoration of pre-injury health and quality of life.

## Mechanisms Impacting Brain Function Following Burn Injury

### Inflammation

Pro-inflammatory cytokines are responsible for the upregulation of inflammation that supports disease progression.^23^ For example, pro-inflammatory cytokines such as tumor necrosis factor alpha are recognized as contributing to pathological pain due to their ability to activate nociceptive sensory neurons, leading to central sensitization.^24^ Following a burn injury, a pro-inflammatory milieu is generated,^5^ which is known to cause systemic changes that can lead to burn survivors developing secondary pathologies such as cardiovascular disease^25^ and, in severe cases, patient death.^26^ In terms of the brain, pro-inflammatory cytokines can affect the permeability of the blood–brain barrier.^27^ Blood–brain barrier hyperpermeability is reported in animal studies, with MRI-derived cerebral edema detected 6 to 18 hours following severe burns on dogs (TBSA = 50%),^28^ and increased MRI T2 relaxation time (marker of tissue water content)^29^ in the hippocampus, thalamus, caudate-putamen, and cerebrum 1 day after generating severe burns on rats (TBSA = 50%).^30^ Diffuse cerebral edema has also been detected in humans within 48 hours of burn injury.^31^ A compromised blood–brain barrier allows peripherally-derived factors like mast cells to enter the brain.^32^ These infringing factors can activate glial cells and T-cells which cause inflammatory and neurotoxic molecules to contribute to neuroinflammation and neuronal apoptosis.^32^ Brain-derived neurotrophic factor (BDNF) expression is significantly reduced by neuroinflammation:^33^ this is important because BDNF is a critical mediator of neural plasticity—the intrinsic ability of the brain to adapt in response to environmental changes, physiological modifications, and experience.^34^ Therefore, it is plausible that the overproduction of pro-inflammatory cytokines following burn injury negatively impacts neural plasticity in burn survivors.

### Afferent Dysfunction

After burn injury, another mechanism that could drive brain functional changes is the inherent damage to the peripheral neural architecture and afferent dysfunction. Time and recovery dependent afferent dysfunction can occur when a burn injury damages cutaneous nerves and their sensory corpuscles that are responsible for providing feedback to the CNS about touch, pain, temperature, and vibration on the skin.^6,18^ Consequently, the brain receives reduced and/or poor quality feedback about external environment interactions at the injured site.^35^ Damaged cutaneous sensory nerves can regenerate and reinnervate the skin, but this is often compromised by scar formation or chaotic wound healing.^36^ It is proposed that scar formation can result in sensory loss due to their lack of sensory adnexal structures, diminished nerve fiber function, and decreased pliability.^35^ Scars can also result in hypersensitivity which is synonymous with pruritus (excessive itch).^37^ Although the mechanisms affecting post-burn pruritis are still debated, the acute mechanisms likely involve pruriceptive and histaminergic pathways which have connections with the thalamus via the spinothalamic tract, and subsequent excitatory connections with the anterior cingulate cortex, insular cortex, and primary and secondary somatosensory cortices.^21,37^ Interestingly, the motor cortex plays an important role in itch processing, including the supplementary motor area, premotor area, and primary motor cortex (M1).^38^ A review of functional magnetic resonance imaging (fMRI) studies on patients with atopic dermatitis highlighted that several observed activation in the motor cortex when participants were restricted from scratching.^38^ It was thus concluded that motor cortex activation could be the result of anticipation to relieve itch symptoms. After the acute recovery phase following burn injury, the histamine-mediated mechanisms of pruritus become less prominent, with chronic pruritus becoming neuropathic due to increases in neuropeptides such as nerve growth factor and substance P and the upregulation of calcium channels in the spinal cord causing central sensitization.^21,37^

Another outcome of severe burn injuries is the destruction of cutaneous sensory nerves that results in the loss of sensory input to the CNS—referred to as deafferentation. Substantial research shows cortical reorganization in response to peripheral injuries that affect sensory function.^39^ For example, fMRI has shown that anterior cruciate ligament injury is associated with diminished activation in several sensorimotor areas and heightened activation in the presupplementary motor area, posterior secondary somatosensory area, and posterior inferior temporal gyrus.^40^ It is thought that the loss of afferent feedback to the CNS from mechanoreceptors located in the ligament is one of the mechanisms driving the brain changes following anterior cruciate ligament injury. Brachial plexus injury can also cause deafferentation and has shown to decrease resting-state interhemispheric functional connectivity between the 2 M1s.^41^ Temporary deafferentation, induced via an ischemic nerve block, has been shown to result in rapid reductions in γ-aminobutyric acid (GABA) concentrations in the sensorimotor cortex^42^ and rapid M1 plasticity.^43^ Changes in brain GABA concentrations play an important role in cortical reorganization following transient changes in sensory input.^42^ Amputation can induce long-lasting M1 reorganization,^44^ which is associated with persistent phantom pain.^45^ Mechanisms that contribute to long-lasting cortical reorganization, such as neurotrophic factors including BDNF, support new synaptic contacts, and axon terminal sprouting.^46,47^ However, BDNF synthesis, release, and action are controlled in an activity-dependent manner,^48^ and thus deafferentation could lead to decreases in BDNF concentrations^49^ and neuronal loss.^50^ Therefore, it is plausible that deafferentation (as well as afferent dysfunction) following burn injuries can induce neural plasticity in the M1,^51^ which might play a role in the lasting motor and sensory dysfunction observed in burn survivors.

### Pain

Burn-induced pain can also induce changes in the CNS due to similar sensitization mechanisms as burn-induced itch.^7^ In fact, the proximity and likely integration and/or association of neural pathways for itch and pain has led researchers to investigate centrally mediated treatment approaches for both.^52^ For burn-induced pain, central sensitization is the result of neuron and circuit function augmentation in nociceptive pathways caused by increases in membrane excitability and decreases in inhibition.^53^ Neural signal amplification causes pain hypersensitivity to a variety of stimuli, including mechanical pressure, chemical substances, light, sound, cold, heat, stress, and electrical stimuli.^53^ As a result, pain no longer acts as a protective response but is triggered when exposed to stimuli which would not invoke tissue damage or injury—a condition referred to as allodynia.^54^ Allodynia is a frequently reported adverse effect following burn injury and has been linked to functional changes in the spinal cord dorsal horn.^55^ Electrophysiology study in rats showed that neurons in the dorsal horn ipsilateral to the burn (partial-thickness burn on hind paw) were hyperexcitable to sensory stimuli, including brush, press, pinch, and serial von Frey filament, within 3 to 7 days of injury.^55^ This hyperexcitability of neurons in the ipsilateral dorsal horn was maximal 4 weeks following injury and persisted for up to 8 weeks following injury. Interestingly, neurons in the dorsal horn contralateral to the burn were hyperexcitable to brush and serial von Frey filament stimuli 4 weeks following injury. The authors suggested that the hyperexcitability in the dorsal horn might be implicated in the development of chronic neuropathic pain following burn injury.^55^ Structural spinal cord changes have also been observed following partial thickness burn injury resulting in persistent pain in mice: Patwa et al^9^ showed increased dendritic spine density on alpha-motor neurons in the ventral horn ipsilateral to a hind paw burn in mice, with the greatest increase in dendritic spines close to the motor neuron soma, which might reflect hyperexcitability.^9^ As dendritic spines play a critical role in normal synaptic transduction, malformed dendritic spines are suggestive of abnormal function.^9^ Given that spinal changes can influence cortical reorganization following peripheral nervous system trauma and disease^56^ it is possible that CNS neuropathology contributes to the chronic pain and dysfunction experienced by burn survivors. Although a full review of spinal changes following burn injury is beyond the scope of the current review, this work undoubtedly contributes to our understanding of the possible mechanisms underlying burn-induced dysfunctions. However, it is worth noting that the descending corticospinal tract of rodents is anatomically and functionally different to humans,^57^ which should be considered when interpreting these findings, and there is ongoing debate regarding the translation of findings from animal models of burn injury to human burn survivors.^58-60^ Recently, MRI cerebral blood volume mapping has shown electrical (TBSA = 6.5%) and non-electrical burn survivors (TBSA = 9.0%) with self-reported chronic pain (~3 months) had plasticity in the sensory pain network: increased cerebral blood volume in the postcentral gyrus and decreased cerebral blood volume in the temporal lobe and insula cortex compared to non-injured controls.^61^ It is possible the cerebral blood volume differences in the sensory cortical regions reflect heightened activity induced by chronic burn pain. These results underpin the links between CNS changes in burn survivors experiencing chronic pain, including cerebral plasticity changes.

## Burn Injury-Related Functional Changes in the Brain

### Motor Cortical Changes Following Burn Injury

A commonly used technique to examine corticospinal excitability and motor cortical plasticity is transcranial magnetic stimulation (TMS). TMS works based on the principle of electromagnetic induction to generate a suprathreshold current in the brain to depolarize neurons in cortical brain regions.^62^ The TMS-induced electrical field can directly activate corticospinal neurons or indirectly activate corticospinal neurons via cortical interneurons as evidenced by the generation of specific descending volleys called direct-waves (D-waves) and indirect-waves (I-waves).^63^ The summation of descending volleys at the spinal level can lead to the firing of α-motoneurons.^64^ Most studies examine the TMS-evoked response by placing EMG surface electrodes over a target muscle to detect the motor evoked potential (MEP) elicited by a suprathreshold TMS pulse: peak-to-peak MEP amplitude provides a measure of corticospinal excitability.^63^

Few studies have used TMS to investigate corticospinal changes following burn injury. Likely the first study to implement TMS to examine burn-induced pathophysiology, Seo et al^65^ compared MEP latency, F-waves, and calculated central motor conduction time (CMCT) outcomes in severely injured burn survivors with burn-induced myelopathy (TBSA = 25.4%) or no myelopathy (TBSA = 27.1%). There was no difference in F-wave latency between the 2 groups in either an intrinsic hand muscle (abductor digiti minimi) or an intrinsic foot muscle (abductor hallucis). There was no difference in MEP latency or CMCT in abductor digiti minimi, however, MEP latency and CMCT were significantly longer in abductor hallucis for the myelopathy compared to the non-myelopathy group. MEP latency represents the corticospinal conduction time (M1 to the muscle), while CMCT represents an estimate of the conduction time of corticospinal fibers between the M1 and spinal motor neurons. The authors concluded that descending central conduction pathway abnormality, detected using TMS, may be useful indicator to diagnose electrical burn-induced myelopathy.^65^ However, it is unclear why central conduction time was slower in the lower limb but not the upper limb in burns survivors with myelopathy compared to burns survivors without myelopathy. Burn location was not reported and, therefore, it is unclear whether burn location affects conduction time. Furthermore, there was no non-burn-injured control group, so it is unclear whether corticospinal conduction is altered in burn survivors compared to non-injured controls.

Garside et al^66^ examined changes in motor cortical inhibition in burn injury survivors by measuring the cortical silent period (cSP) duration. cSP duration reflects the suppression of descending drive from the M1 and subsequent disfacilitation of the motoneurons^67^ and is thought to be mediated at the cortical level by both GABA receptor classes (GABA_a_ and GABA_b_).^68^ Garside et al^66^ delivered single-pulse TMS to the M1 during active contraction of an intrinsic hand muscle (first dorsal interosseous) to examine cSP in burn survivors and non-injured controls. Overall, there was no significant difference in cSP duration between the 2 groups. However, the burn survivor sample was heterogenous, and exploratory analyzes of burn survivor subgroups showed that the cSP duration was shorter on the burn-affected side compared to non-injured controls for burn survivors who: (i) had an upper-limb burn, (ii) were <2 years post burn injury, (iii) had a burn <10% TBSA, and (iv) had a partial thickness burn. These results provided preliminary evidence to suggest that a burn injury can reduce motor cortical inhibition.

Whife et al^69^ used a paired-pulse TMS technique to examine the excitability of M1 intracortical inhibitory circuits in burn survivors and non-injured controls. In paired-pulse TMS, when a subthreshold conditioning stimulus precedes a suprathreshold test stimulus by 1 to 6 ms, the resultant MEP amplitude is suppressed.^70^ The conditioning stimulus activates low-threshold inhibitory interneurons that generate synaptic inhibition of corticospinal neurons.^71^ As a result, the impact of GABA_a_-mediated intracortical inhibition on the suprathreshold test stimulus can be specifically assessed by comparing the MEP amplitudes between the single- and paired-pulse protocols, known as short-interval intracortical inhibition (SICI).^72^ Whife et al^69^ examined SICI in burn survivors 6 and 12 weeks following their injury and in non-injured controls 6 weeks apart. Results showed no differences in SICI between the burns survivors and non-injured controls suggesting no change in GABA_a_-mediated intracortical inhibition up to 12 weeks following burn injury. However, SICI was only assessed at a single conditional stimulus intensity (80% of resting motor threshold) and a single interstimulus interval (2 ms): a more comprehensive examination of SICI using multiple conditioning stimulus intensities and multiple inter-stimulus intervals is required to conclusively determine whether GABA_a_-mediated intracortical inhibition is altered following burn injury. Moreover, although the SICI result from Whife et al^69^ appears somewhat inconsistent with the cSP findings reported by Garside et al,^66^ pharmacological studies show that SICI provides a measure of GABA_a_-mediated intracortical inhibition, while cSP provides a measure of both GABA_a_-mediated and GABA_b_-mediated intracortical inhibition.^68^ Another paired-pulse TMS technique called long-interval intracortical inhibition (LICI), where 2 suprathreshold pulses are delivered 100 to 200 ms apart, provides a measure of GABA_b_-mediated intracortical inhibition.^73^ Future work should measure LICI in burn survivors to better understand burn-induced excitability changes in M1 GABA_b_-mediated intracortical inhibitory circuits.

A recent study using repetitive TMS (rTMS) has investigated the motor cortical plasticity capacity of burn survivors compared to non-injured controls.^8^ rTMS can modulate neuron excitability by delivering trains of TMS pulses to targeted brain regions.^74^ The frequency, intensity, and pattern of stimulation can affect the direction of the neuronal excitability change, with specific rTMS protocols shown to increase or decrease cortical excitability.^75^ The directional changes in corticospinal excitability following rTMS are thought to reflect long term potentiation-like and long term depression (LTD)-like plasticity based on the notion that TMS-induced plasticity occurs due to synaptic strengthening or weakening.^76^ More recently, researchers have been examining plasticity capacity by administrating a pattern of magnetic pulses called theta burst stimulation. The primary theta burst stimulation protocols are intermittent theta burst stimulation and continuous theta burst stimulation (cTBS) which are expected to increase and decrease corticospinal excitability, respectively.^75^ Whife et al^8^ examined cTBS-induced motor cortical plasticity capacity in burn survivors. At 6 weeks following burn injury, there was no change in MEP amplitude following 2 applications of cTBS in burn survivors but the expected decrease in MEP amplitude was observed in non-injured controls; at 12 weeks following injury, there were no differences in change in MEP following 2 applications of cTBS in burns survivors and non-injured controls. Importantly, the researchers also demonstrated a significant association between cTBS-induced neuroplasticity 12 weeks after burn injury and functional outcome scores in the SF-36 survey: those burn survivors who showed the greatest LTD-like neuroplasticity following cTBS (expected response) showed the highest functional outcome scores.^8^ These results indicate that burn survivors have reduced LTD-like neuroplasticity capacity at the early phase of their recovery and provide preliminary evidence that M1 plasticity might be associated with better physical function recovery in burn survivors.

Transcranial direct current stimulation (tDCS) has also been used to induce M1 plasticity in burn survivors’.^77^ tDCS modulates the excitability of cortical neurons by sending weak electrical currents from an anode to a cathode placed on the scalp.^78^ Excitability of the brain region underneath the anode is increased and excitability of the brain regions underneath the cathode is decreased. Portilla et al,^77^ targeted the M1 with a single session of anodal tDCS in 3 patients experiencing chronic neuropathic-like pain after burn injury and showed that anodal tDCS decreased single-pulse MEP amplitude and increased intracortical inhibition compared to sham stimulation. Although these results conflict with typical responses detected in healthy participants,^79^ they are consistent with other studies that investigated TMS changes in chronic pain conditions such as fibromyalgia and rheumatoid arthritis.^80^ These results highlight the novelty and potential importance of targeting the brain for burn rehabilitation as these atypical responses may reflect maladaptive cortical plasticity following burn injury. However, interpretation of these results should be treated with caution given the small sample size.

### Non-Motor Cortex Changes Following Burn Injury

One of the first studies to systematically investigate brain function changes following burn injury used electroencephalography or electroencephalogram (EEG).^81^ EEG is one of the most frequently used non-invasive techniques to assess brain electrophysiological dynamics as well as linking those findings to disease.^82^ Although interpretation of EEG waveforms can be challenging, the most common approach is to characterize EEG waveforms in defined frequency bands: delta (0.5-4 Hz); theta (4-7 Hz); alpha (8-12 Hz); sigma (12- 16 Hz); and beta (13-30 Hz).^83^ The frequency bands are associated with various physiological states such as sleep and wake states but abnormalities can represent cerebral dysfunction.^84^ Thus, EEG has been used to support burn encephalopathy diagnosis, including seizure symptoms. Several earlier studies detected what was classified as “non-specific” EEG abnormalities following burn injury, meaning these findings did not support a specific diagnosis such as seizures. For example, Petersén^81^ reported diffuse (spread over large areas of both sides of the head) or focal slow wave rhythmic abnormalities in occipital and frontal-temporal regions in more than half of the burn survivors sampled. The generalized background slowing of EEG waveforms usually occurs in the theta or delta bands^84^ and reflects global cerebral dysfunction, while focal slowing reflects cerebral dysfunction of the underlying brain region.^85^ Hughes et al,^86^ who used EEG to test for abnormal spiking activity associated with seizures in acute and chronic burn survivors, showed either diffuse or focal abnormalities in either slow wave, spiking, or both, and that at least some changes were recorded at centrally aligned electrodes, indicating changes in the excitability of motor areas. Importantly, the EEG abnormality prevalence was greatest in burn survivors who suffered more severe burns (TBSA > 20%). Generalized slowing of EEG waveforms has also been detected in children (4 out of 6 tested) diagnosed with burn encephalopathy (TBSA > 30%).^87^ Interestingly, Hughes et al^86^ observed that EEG abnormalities peaked appropriately 3 to 11 days following burn injury which coincides when metabolic aberration and death tends to occur.^88^ However, the EEG abnormalities would generally improve 2 weeks after injury.^86^ It should be mentioned that although these earlier EEG findings have led to important further research into functional brain changes following burn injury, current EEG methodologies involving data acquisition and analysis are quite different. Therefore, researchers should temper expectations of conclusive outcomes when relying on these findings.

More recently, Miraval et al^89^ reported that a sample of 4 burn survivors with chronic itch (>1 year) had decreased alpha and beta power in the occipital and frontal regions of the brain, respectively, when compared to healthy controls. Although these preliminary findings demonstrate that altered neural oscillations occur in burn survivors with chronic itch, this conclusion should be treated with caution due to the small sample tested. Moreover, it is unclear what electrodes were included in their regional assessment, with it possible that centrally aligned electrodes (ie, motor areas) were included in their frontal region definition. Other researchers have since found no global difference in oscillatory power and peak frequency across theta, alpha, and beta frequency bands using continuous EEG between 15 burn survivors with chronic itch and 15 healthy controls.^90^ These findings were seen during resting and stimulation (induced itch) conditions of non-injured skin, and thus, it was concluded that the mechanisms influencing itch symptoms in burn survivors may be mediated by local factors as opposed to involving central cortical processing. However, more research is needed to investigate the brain electrophysiological dynamics of burn-related symptoms as greater insight may highlight the mechanisms underlying persistent functional impairments.

Non-motor cortex changes following burn injury can also be investigated using other techniques. For example, functional near-infrared spectroscopy can highlight changes in cortical hemodynamics due to its ability to detect changes in oxygenated and deoxygenated hemoglobin concentrations in the targeted cerebral cortex.^91^ A recent study demonstrated that prefrontal cortex activation during walking was significantly greater in burn survivors with severe lower body injuries compared to those with upper body injuries and healthy controls.^92^ It is possible that movements involving body parts affected by burns require greater cognitive resources than usual. Although this study highlights the utility of implementing functional near-infrared spectroscopy to detect cortical activity as a result of burn injury, more research is required to validate these findings in a larger burn survivor cohort.

## Targeting the Brain to Support Treatment, Rehabilitation, and Recovery

Researchers have been examining the efficacy of treatment and rehabilitation approaches in burn survivors that are thought to induce neural plasticity. If functional changes in M1 following burn injury are associated with better motor and sensory outcomes, it is possible that targeting M1 in burn rehabilitation might reduce motor and sensory dysfunction. Although non-invasive brain stimulation (NIBS) treatment effectiveness is still contested,^76^ the consensus is it can induce positive effects in stroke survivors such as decreasing spasticity and improving motor function.^93^ One between-subject study examined the effects of repeated tDCS application to M1 in burn survivors and observed no change in pain and itch levels following 10 and 15 sessions of anodal tDCS, while sham stimulation improved itch levels at the 2-week follow-up, indicating a placebo effect.^52^ The researchers suggested that the targeting of M1 may not be ideal to treat pain and itch symptoms and suggested that targeting the prefrontal cortex, where regulation of affective conditions occurs, may be more beneficial in the future. It is also possible that tDCS treatments would be more effective in acute burn survivors than chronic burn survivors for pain and itch outcomes, with prolonged burn-induced dysfunctions becoming more resilient to treatments: Thibaut et al^52^ recruited burn survivors’ years after their injury (tDCS: 7.2 ± 7.3 year vs sham tDCS: 11.5 ± 10.5 year). Other researchers have targeted the sensory cortex using a single session of cathodal tDCS and found that pain state anxiety immediately after wound dressing (measured with self-reported questionnaires) was significantly reduced in burn survivors who received cathodal tDCS compared to sham stimulation.^94^ This result suggests that targeting sensory regions of the brain with tDCS that decreases excitability may offer some pain anxiety relief in burn survivors. It is important for future research to examine whether repeated exposures of cathodal tDCS targeting the sensory cortex can also reduce pain and itch, including chronic symptoms.

It should be noted that researchers and clinicians have been implementing other interventions that are thought to drive neural plasticity for burn treatment and rehabilitation. For example, hypnotherapy has been successfully implemented for decades to attenuate pain and anxiety in burn patients, with hypnosis thought to inhibit activity within fear circuits in the brain.^95^ More recently, virtual reality has been used to attenuate pain by distracting burn patients during wound care and physiotherapy treatments^96^. It is recognized that distraction tasks alleviate pain by drawing attentional focus away from the processing of pain.^97^ In participants who received thermal pain stimuli, the pain relief benefits of virtual reality were associated with blood oxygen level dependent fMRI changes in the anterior cingulate cortex, the primary and secondary somatosensory cortices, the insula, and the thalamus.^98^ With it above mentioned that the postcentral gyrus (ie, primary somatosensory cortex) and insula cortex show plasticity effects in burn survivors with chronic pain,^61^ the findings demonstrate that virtual reality can influence the function of brain regions associated with burn-induced pain. For physiotherapy treatments, the pain relief could increase engagement and movement proficiency, leading to greater benefits from physical rehabilitation.

## Recommendations for Future Research

A better understanding of the neurophysiological changes that occur following burn injury would provide an evidence base for targeting the brain for burn injury rehabilitation. For example, research is needed to understand the functional brain changes in acute and chronic burn survivors as well as determine whether treatments that target brain function support burn recovery. The paucity of research exploring brain function following burn injury, including understanding maladaptive changes, could be limiting our understanding of the mechanisms underpinning persistent burn-induced symptoms. Although the positive effects of NIBS treatments have been determined in other clinical populations such as stroke survivors more research is needed to determine the prescription parameters and exposure timing to reliable treat burn survivors. Implementing protocols that seek to meaningfully alter brain function and ultimately, facilitate neural plasticity and/or prevent maladaptive neural changes are the primary aims. For example, it is plausible that NIBS treatments targeting brain regions associated with reported symptoms might be effective at improving outcomes such as repeated application of NIBS targeting the prefrontal cortex to treat pain and M1 to treat motor impairment. In using NIBS to treat burn-induced symptoms, it will be critical to consider the direction of excitability change of the targeted cell populations. NIBS techniques can increase or decrease the excitability of targeted cells^76^ and, therefore, basic neurophysiological research is required to determine whether facilitatory or inhibitory NIBS protocols applied to target brain regions would be most beneficial for functional improvements. For example, the M1 may benefit from facilitatory treatments to support physical rehabilitation, while the prefrontal cortex, especially regions associated with pain, may benefit from inhibitory treatments. Future research examining the efficacy of NIBS on functional recovery following burn injury must also consider adherence to the intervention in this population. Indeed, 1 study examining the efficacy of tDCS to reduce pain and itch required 23 visits from burn survivors and reported a dropout rate of 45%.^52^ Consumer engagement is required to identify barriers and facilitators to participating in such interventions among burn survivors.

In addition to the application of NIBS alone for neurorehabilitation after burn injury, it is likely the combination of NIBS interventions and existing treatment approaches such as physiotherapy will lead to better functional outcomes for burn survivors than either intervention applied in isolation. In particular, the implementation of NIBS techniques that seek to increase M1 excitability such as intermittent theta burst stimulation before physiotherapy may prime the M1 and thus, support greater physical rehabilitation as is the case in stroke rehabilitation.^99^ The changes observed in the cortex and spinal cord also warrant further investigation to fully understand the responses to burn injury in the defined anatomical compartments of the CNS. With more basic research identifying the neural correlates of burn-induced motor and sensory dysfunction such as pain there will be a strong neurophysiological evidence base for large, randomized controlled trials, which could ultimately underpin a change in clinical practice for burn injury.

## Conclusion

Given the high prevalence of non-severe burns requiring non-surgical treatments, more research is needed to better understand the neurophysiological changes following burn injury as well as the effectiveness of non-invasive CNS treatments to support burn rehabilitation and recovery. There is sufficient evidence to suggest that neurophysiological changes do occur following burn injury, and a significant association between the capacity for neural plasticity and self-reported functional scores, suggests that NIBS treatments may provide improved outcomes. Understanding and directing early, functionally beneficial neural plasticity following burn injury could reduce maladaptive plasticity and attenuate poor pain and functional outcomes.
